# The dynamic changes in autophagy activity and its role in lung injury after deep hypothermic circulatory arrest

**DOI:** 10.1111/jcmm.17165

**Published:** 2022-01-11

**Authors:** Minjian Kong, Dongdong Wei, Xuebiao Li, Xian Zhu, Ze Hong, Ming Ni, Yifan Wang, Aiqiang Dong

**Affiliations:** ^1^ Department of Cardiovascular Surgery The Second Affiliated Hospital of Zhejiang University School of Medicine Hangzhou China

**Keywords:** autophagy, deep hypothermic circulatory arrest, ischaemia/reperfusion, lung injury

## Abstract

Deep hypothermic circulatory arrest (DHCA) can cause acute lung injury (ALI), and its pathogenesis mimics ischaemia/reperfusion (I/R) injury. Autophagy is also involved in lung I/R injury. The present study aimed to elucidate whether DHCA induces natural autophagy activation and its role in DHCA‐mediated lung injury. Here, rats were randomly assigned to the Sham or DHCA group. The sham group (*n* = 5) only received anaesthesia and air intubation. DHCA group rats underwent cardiopulmonary bypass (CPB) followed by the DHCA procedure. The rats were then sacrificed at 3, 6 and 24 h after the DHCA procedure (*n* = 5) to measure lung injury and autophagy activity. Chloroquine (CQ) was delivered to evaluate autophagic flux. DHCA caused lung injury, which was prominent 3–6 h after DHCA, as confirmed by histological examination and inflammatory cytokine quantification. Lung injury subsided at 24 h. Autophagy was suppressed 3 h but was exaggerated at 6 h. At both time points, autophagic flux appeared uninterrupted. To further assess the role of autophagy in DHCA‐mediated lung injury, the autophagy inducer rapamycin and its inhibitor 3‐methyladenine (3‐MA) were applied, and lung injury was reassessed. When rapamycin was administered at an early time point, lung injury worsened, whereas administration of 3‐MA at a late time point ameliorated lung injury, indicating that autophagy contributed to lung injury after DHCA. Our study presents a time course of lung injury following DHCA. Autophagy showed adaptive yet protective suppression 3 h after DHCA, as induction of autophagy caused worsening of lung tissue. In contrast, autophagy was exaggerated 6 h after DHCA, and autophagy inhibition attenuated DHCA‐mediated lung injury.

## 1 INTRODUCTION

The deep hypothermic circulatory arrest (DHCA, a type of cardiopulmonary bypass (CPB)) procedure is required in certain cases undergoing aortic arch surgery and complex congenital heart surgery, as it not only increases the body's tolerance to the harsh environment of ischaemia and hypoxia due to a decrease in tissue metabolism and oxygen consumption but also provides a relatively bloodless surgical field, thus increasing the likelihood of a successful surgery.^1^ Although continuous improvements in DHCA technology and CPB circuit materials have significantly reduced postoperative complications and mortality, approximately 20%–30% of patients undergoing CPB with DHCA still experience varying degrees of postoperative pulmonary dysfunction, ranging from transient lung injury in mild cases to acute respiratory distress syndrome in severe cases.^2^, ^3^ The incidence of acute respiratory distress syndrome after cardiac surgery related to CPB is 0.4%–1.3%, and mortality rates of 15%–68.4% are reported among these patients.^4^, ^5^ The underlying mechanisms for acute respiratory distress have been suggested to be multifactorial; in fact, its related pathological process primarily recapitulates that of ischaemia/reperfusion (I/R)‐induced lung injury^6^, ^7^ and systemic inflammatory response syndrome.^8^

As an essential biological process for maintaining normal cell homeostasis, autophagy plays key roles not only in physiological states of the lung tissue^9^ but also in various pathophysiological processes of lung injury,^10^, ^11^, ^12^ including I/R‐induced lung injury.^6^, ^7^ Different signalling pathways are involved in and contribute to lung injury, such as the mTOR/TLR4/NF‐κB,^13^ ERK 1/2,^14^ and MAPK and NF‐κB^15^ signalling pathways. Intriguingly, autophagy can exert either beneficial (or protective)^14^, ^16^ or detrimental effects^7^ on lung tissue during the pathological process of reperfusion injury, and these effects depend on the stage and severity of a specific disease, which are closely related to dynamic microenvironmental conditions.^17^, ^18^ On the other hand, studies have been performed to elucidate the underlying mechanisms of DHCA‐mediated lung injury^19^, ^20^, ^21^; however, few studies have focused on the role of autophagy in DHCA‐mediated lung injury. Therefore, the aims of the present research were (a) to investigate whether DHCA induces time course damage in lung tissue and whether this damage was associated with dynamic changes in autophagy activity, (b) to quantify phase‐dependent changes in autophagic flux and finally (c) to evaluate whether manipulation of autophagy activity affects DHCA‐mediated lung injury.

## 2 METHODS

We declare that all data that support the findings of this study are available within the article and its Appendix S1.

A more detailed description of the experimental methods (including the human bronchial epithelioid cell line 16HBE cells culture, 16HBE cells in vitro oxygen glucose deprivation (OGD)/re‐oxygenation (OGD/R) model, coculture of 16HBE cells and serum of experimental rats, LC3 immunofluorescence staining of 16HBE cells and terminal deoxynucleotidyl transferase dUTP nick end labelling (TUNEL) assay) is available in the Appendix S1 (private link: https://figshare.com/s/9d775fb577d553c5e7c5, DOI: https://doi.org/10.6084/m9.figshare.14938407.v1).

### 2.1 Animals

Twelve‐week‐old male Sprague‐Dawley (SD) special pathogen‐free rats weighing between 400 g and 450 g (the Slaccas Animal Center, Shanghai, China) were studied. The animal research study protocol complied with the *Guide for the Care of Use of Laboratory Animals* published by the National Institutes of Health (NIH Pub. No. 85‐23, revised 1996) and was approved by the Institutional Animal Care and Use Committee of Zhejiang University School of Medicine. All rats were housed in an animal house under controlled conditions and allowed free access to tap water and a standard diet for 2 weeks as an acclimatization period before the experiments. Staff regularly disinfect facilities, including cage boxes, food boxes, water bottles, drinking spouts, etc.

### 2.2 Surgical preparation

The detailed methods for the rat model of DHCA have been described in our previous report with minor modifications.^22^ Briefly, 12‐week‐old rats were anaesthetized with sodium pentobarbital (50 mg/kg) by intraperitoneal injection (i.p.) and endotracheally intubated with a 16‐gauge cannula, which was then connected to an animal respirator (Model alc‐v8s, ALCOTT, Shanghai). Anaesthesia was maintained with 1.5%–2.0% sevoflurane (Hengrui Pharmaceutical, Shanghai, China) ventilation and mechanical ventilation was performed with a tidal volume of 8 ml/kg and at a respiratory rate of 60 cycles per minute,^23^ both of which were adjusted according to blood gas analyses obtained at five time points. The left superficial femoral artery, right external jugular vein and tail artery were dissected. Using a surgical approach, the left superficial femoral artery was cannulated with a preheparinized 24‐gauge catheter for both the delivery of heparin sodium (500 IU/kg, H32022088, Qianhong Biopharma, Changzhou, China) to perform systemic anticoagulation and facilitate connection to a multidirectional physiological monitor (Powerlab, Harvard Apparatus, America) to continuously monitor both mean artery pressure (MAP) and heart rate (HR). The right external jugular vein was also cannulated with a homemade 24‐side venous drainage tube to serve as a venous outflow. The median tail artery was catheterized using a 20‐gauge catheter, which served as the arterial inflow for the CPB circuit. Rectal temperature was continuously monitored (Powerlab, Harvard Apparatus, America).

### 2.3 Execution of CPB and DHCA

**FIGURE 1:**
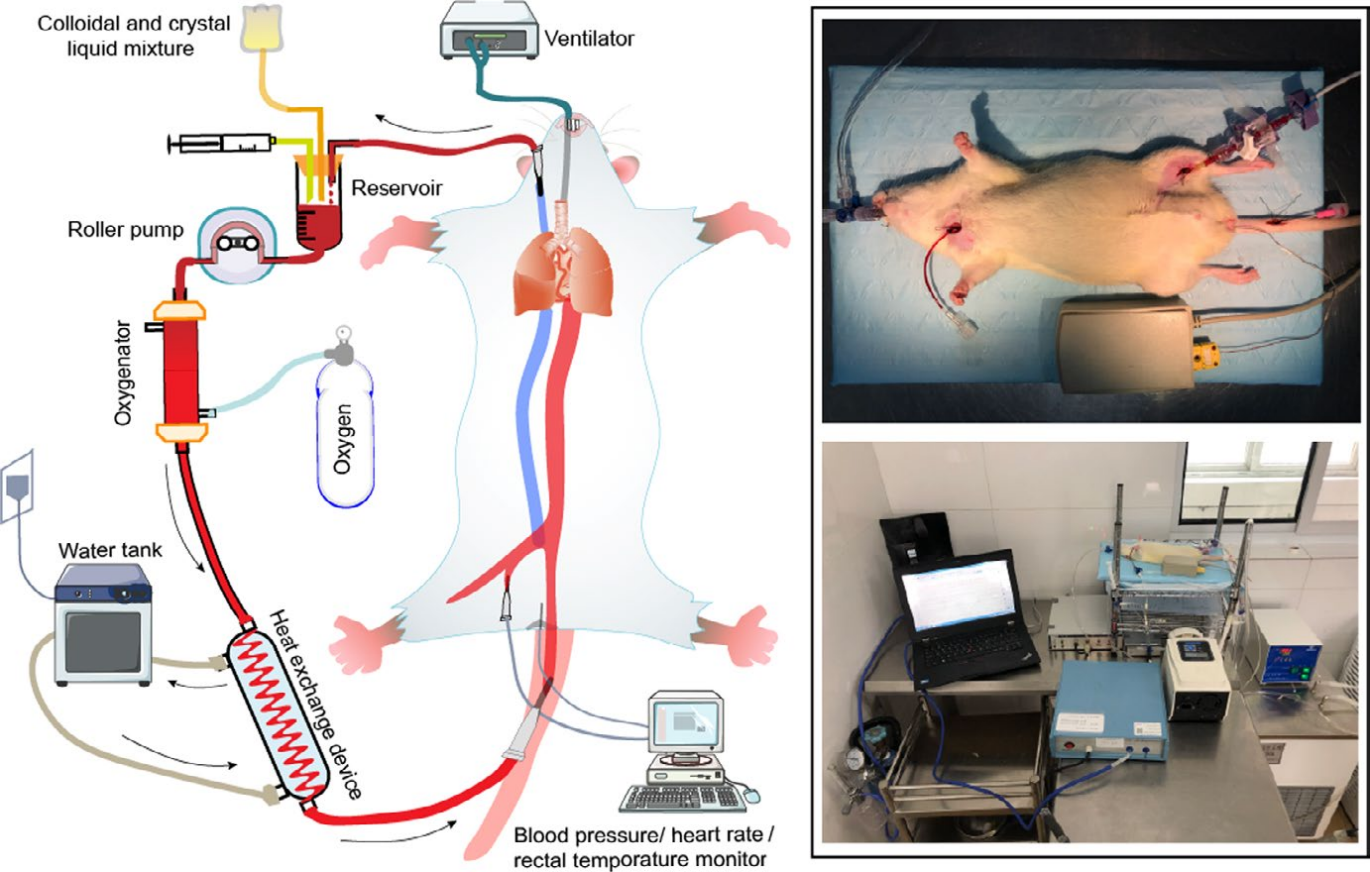
The Schematic Diagram of Our Model Establishment. The right external jugular vein, left superficial femoral artery and tail artery were catheterized. The cardiopulmonary bypass device mainly includes a venous reservoir, roller pump, membrane oxygenator and heat exchanger. Blood from the jugular vein was drained into the venous reservoir from which the blood was then, with the aid of a roller, pumped into the membrane oxygenator for gas exchange. A heat exchanger followed before finally entering the body through the caudal artery. Mean arterial pressure, heart rate anal temperature, and other parameters were continuously monitored

The CPB device (for details, Figure 1) is mainly composed of a venous reservoir, roller pump (bt100‐2j, Lungerpump, Shanghai, China), membrane oxygenator (Micro‐MO, Kewei, Guangdong, China) and heat exchanger customized for rat experiments (Xijing Medical Products Incorporation, Xi'an, China). The circuit was primed with 8 ml of 6% hydroxyethyl starch (H20103246, Fresenius Kabi, Beijing, China). Blood from the jugular vein was drained into the venous reservoir from which the blood was immediately pumped into the membrane oxygenator with the aid of a roller for gas exchange. A heat exchange process then followed before the blood finally entered the body through the caudal artery. During CPB, the flow rate started at 135 ml/kg/min and gradually decreased during the cooling phases.

Once the CPB was established, systemic cooling was initiated via a heat exchanger assisted by ice bags. Controlled cooling was maintained for 30 min, and ventilation was maintained at a lower tidal volume and frequency, which was adjusted based on the blood gas analysis at the dedicated time points described above. When the rectal temperature reached 18°C, CPB and ventilation were completely interrupted to generate a 50‐min circulatory arrest, that is, all the organs of the rats were exposed to ischaemia. Once the circulation arrest period was completed, the CPB was restarted. The rats were rewarmed with a heated blanket to ensure that a rectal temperature of 35°C was reached within 30 min. Once the rectal temperature was stable at 35°C, CPB and anaesthesia were terminated, whereas ventilation was maintained for an additional 60 min, allowing for full recovery from anaesthesia. The remaining priming solution was infused if needed to maintain a MAP greater than 80 mmHg.

To maintain stable homeostasis, arterial blood gas analysis was regularly sampled and analysed at the following five time points, which were selected based on previous reports both from other^23^ and our own laboratory^22^: 10 min pre‐CPB (T1), precardiac arrest (T2), cardiac rebeating (T3), weaning of CPB (T4) and before sacrifice (T5).

### 2.4 Experimental protocols

#### 2.4.1 Protocol I

To assess whether DHCA can induce lung injury, 20 SD rats were randomly assigned and divided into 4 groups (*n* = 5). In the sham group (S group), all rats underwent anaesthesia, air intubation, ventilation and vascular insertion of the cannulas without standard CPB and DHCA procedures. The other three groups included DHCA 3 h group (D3 h group), DHCA 6 h group (D6 h group) and DHCA 24 h group (D24 h group), each of these group rats experienced standard CPB and DHCA intervention as described above and were sacrificed at 3 h, 6 h and 24 h after weaning of CPB respectively. These time points were determined based on previous reports from other groups^24^, ^25^ and our own results.^22^ The extent of lung injury, including lung histology examination, wet/dry lung weight ratio and inflammatory cytokine quantification, was assessed for each group of rats.

#### 2.4.2 Protocol II

To describe the dynamic changes in autophagy activities in the lung tissue obtained from the rats subject to *Protocol I* (including the S, D3 h, D6 h and D24 h groups), the anterior lobe and middle lobe of the right lung were used for Western blotting to quantify the protein expression levels of LC3‐II, Beclin 1, ATG5 and p62, and immunofluorescence staining for LC3‐II was also performed.

#### 2.4.3 Protocol III

To further assess the dynamic details of the autophagy process, autophagic flux was also evaluated using chloroquine (CQ, C6628, Sigma Aldrich, America, 20 mg/kg in PBS, i.p.). The D3 h and S group rats were given CQ 2 h before euthanization to assess the autophagic flux at the early phase after the DHCA procedure, and similarly, CQ was also delivered to the other two groups (including the D6 h and S groups) rats hours before the end of the experiments to assess the late phase changes in autophagic flux after DHCA intervention.

#### 2.4.4 Protocol IV

To assess whether activation (rapamycin, V900930, Sigma Aldrich, America, 4 mg/kg in 1.8% DMSO + PBS, i.p.) or suppression (3‐methyladenine, 3‐MA, M9281, Sigma Aldrich, America, 30 mg/kg in PBS, jugular vein injection) of autophagic activity can modulate the lung injury induced by the DHCA procedure. Rapamycin or 3‐MA was delivered to either the D3 h group or the D6 h group (with their corresponding solvents serving as the placebo for a control group respectively) to further delineate whether autophagy plays protective or detrimental roles in lung injury induced by the DHCA procedure.

### 2.5 Animal euthanization and tissue handling

Once each experimental protocol was completed, the rats from each group were sacrificed with an overdose of anaesthesia (sodium pentobarbital, 100 mg/kg (i.p.)), and the lung tissue was obtained. The right lung tissue was processed for the following studies: (1) The posterior lobe of the right lung was used to collect bronchoalveolar lavage fluid (BALF) for cytokine quantification; (2) Most of the anterior lobe and middle lobe of the right lung were harvested and stored in liquid nitrogen for Western blot, and some of these tissues were also used for transmission electron microscopic examination. The left lung tissue was also harvested. A portion of the left lung tissue was immediately fixed in 10% neutral formalin solution (AG2160, ACMEC Biochemical, Shanghai, China) and paraffin‐embedded for haematoxylin and eosin staining and immunofluorescence staining, and the remaining portion of the left lung was used to assess the wet/dry weight ratio. Blood samples were collected for cytokine quantification.

### 2.6 Histological assessment of lung injury

As described above, a portion of the left lung was immediately fixed in 10% neutral formalin solution (AG2160, ACMEC Biochemical, Shanghai, China) and paraffin embedded for haematoxylin and eosin staining. Images were obtained using an Olympus BX53 inverted microscope (Olympus, Melville, NY). Three uninformed investigators performed the histological analysis in a blinded fashion, and the final score was determined by the average value of the data obtained from the three independent investigators. The severity of lung injury was determined by quantifying the following four parameters: (I) interstitial oedema, (II) infiltration of inflammatory cells, (III) alveolar haemorrhage and (IV) alveolar septal congestion.^23^ A score for each parameter on a scale of 0–4 is obtained to represent the different levels of severity: 0, for no or very minor; 1, for modest and limited; 2, for intermediate; 3, for widespread or prominent; and 4, for widespread and most prominent.^26^ The total histopathological score was expressed as the sum of all four parameters.

### 2.7 Quantification of cytokines in blood serum and bronchoalveolar lavage fluid

The lung tissues obtained from each group of rats as described above were individually removed and placed in disposable Petri dishes. The bronchi were fixed with forceps, and a 1‐ml syringe with a 20‐gauge indwelling needle was inserted into the bronchi and ligated to prevent overflow during lavage. The lavage of the right lower lung lobe was repeated thrice with 1 ml of 4°C PBS (C0221A, Beyotime, Shanghai, China), and the BALF was collected. The collected fluid (approximately 0.8 ml) was kept on ice and then centrifuged at 4°C (400 g for 5 min). The supernatant was stored at −80°C for further analysis. The total protein concentrations in the supernatant of BALF were determined using the bicinchoninic acid (BCA) protein assay kit (23227, Thermo Fisher Scientific, Shanghai).

Blood samples were collected immediately after the rats were euthanized and kept on ice. The blood samples were centrifuged at 1000 g for 20 min at 4°C, and the serum was collected and stored for further analysis.

The protein levels of tumour necrosis factor α (TNF‐α), interleukin‐6 (IL‐6), myeloperoxidase (MPO) and intercellular cell adhesion molecule‐1 (ICAM‐1) in serum and BALF were measured using ELISA kits (MultiSciences, Hangzhou, China).

### 2.8 Lung wet/dry weight ratio

As described above, a portion of the left lung was weighed after removing superficial blood (wet weight, W). The lung samples were then dried in an oven (at 58°C) for 48 h, and weighed to obtain the dry weight (D). The ratio of wet lung weight to dry lung weight was calculated (W/D).

### 2.9 Western blot

The anterior lobe and middle lobe of the right lung were obtained as described above and stored in liquid nitrogen for protein quantification by Western blot. The tissues were homogenized and lysed in RIPA lysis buffer. The protein concentration was measured by BCA assay (23235, Invitrogen), and an equal amount of protein from all samples was subjected to gel electrophoresis using an 8%‐15% sodium dodecyl sulphate‐polyacrylamide gel (SDS‐PAGE) and then transferred to a polyvinylidene fluoride (PVDF) membrane. PVDF membranes were blocked with either 5% (w/v) nonfat milk in PBS containing 0.1% (v/v) Tween‐20 for 1 h and then incubated with the indicated primary antibodies overnight at 4°C followed by secondary antibodies conjugated with horseradish peroxidase. Immunocomplexes were visualized using a chemiluminescence Western blot detection system (BioRad). The primary antibodies used were as follows: LC3‐I/II (4108, Cell Signaling Technology, Shanghai), Beclin 1 (11306‐1‐AP, Proteintech, America), ATG5 (10181‐2‐AP, Proteintech, United States of America) and SQSTM1/p62 (ab91526, Abcam, Shanghai).

### 2.10 Transmission electron microscopy

The lung tissues from the indicated group rats as described above were prepared for transmission electron microscope examination following the standard methods as described previously.^27^ Briefly, samples approximately 1 mm^3^ in size were fixed with 2.5% glutaraldehyde (R20510, Yuanye, Shanghai, China), postfixed in osmium tetroxide, stained with uranyl acetate, dehydrated with ethanol solutions and embedded in epoxy resin. Thereafter, the blocks were trimmed and cut into ultrathin sections (120 nm) of the indicated thickness. The sections were subsequently observed under a transmission electron microscope. Images were obtained using a TECNA1 10 transmission electron microscope (FEI, Hillsboro, OR, America).

### 2.11 Immunofluorescence staining

The lung tissues obtained from group rats that were designated for haematoxylin and eosin staining were fixed in 10% neutral formalin, paraffin embedded and sliced (3 microns). After deparaffinization and antigen retrieval, the sections were blocked with 3% BSA reagent (A8020, Solarbio, Beijing) for 20 min to avoid nonspecific binding followed by incubation with a primary anti‐LC3B antibody (ab63817, Abcam, Shanghai) overnight at 4°C in a wet box. The tissue slices were then washed thrice with PBS‐T (1× PBS 0.1% Tween 20) and incubated with secondary antibody (5488S, red, Cell Signaling Technology, Shanghai) at 25°C for 1–2 h in a black wet box. Finally, sections were washed and incubated with antifade mounting medium with DAPI (S2110, Solarbio, Beijing) for 30 min. Images were acquired under a fluorescence microscope (IX51, Olympus, Japan).

### 2.12 Statistical analysis

Data with normal distribution confirmed by Kolmogorov‐Smirnov method were presented as mean ± standard deviation. One‐way ANOVA followed by Tukey's honest significant difference (HSD) post hoc test was used for comparison between at least three independent groups, including the levels of inflammatory factors (TNF‐α, IL‐6, MPO, ICAM‐1), the expression levels of autophagy‐related proteins (LC3‐II, Beclin 1, ATG5 and p62), the number of LC3‐positive cells in IF. Nonparametric Mann‐Whitney U test was used for lung injure score. All data were analysed using SPSS (version 17.0) statistical software. A *p* value <.05 was considered statistically significant.

## 3 RESULTS

### 3.1 Time‐dependent lung injury secondary to DHCA procedure

**FIGURE 2:**
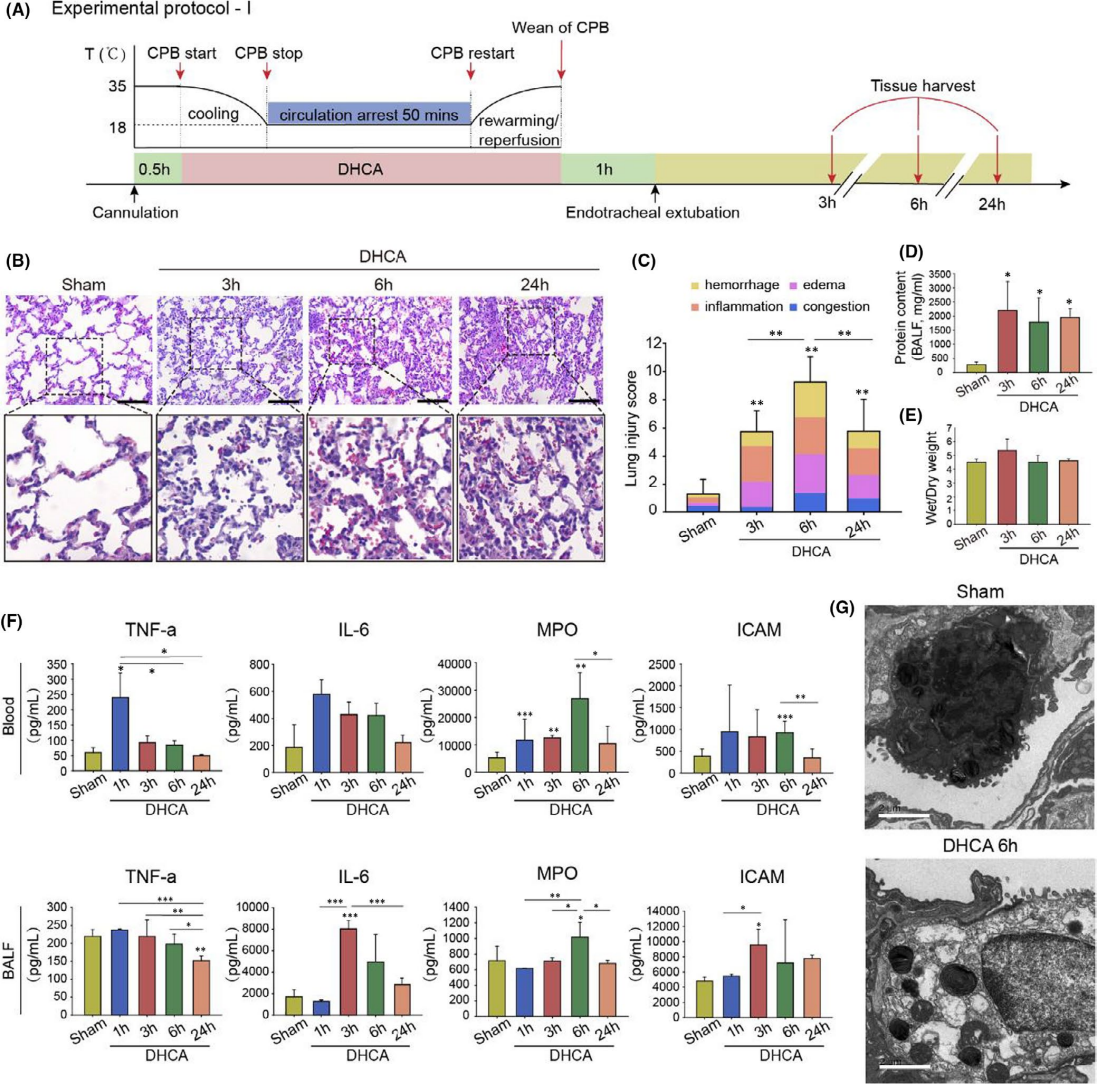
Time‐dependent Lung Injury Secondary to Deep Hypothermic Circulatory Arrest (DHCA) Procedure. (A) Experimental Protocol I. Twenty SD rats were randomly divided into four groups: Sham group, in which all rats underwent anaesthesia, air intubation, ventilation and vascular insertion of the cannulas without standard CPB and DHCA procedures, and DHCA 3 h group, DHCA 6 h group and DHCA 24 h group for which rats experienced standard CBP and DHCA intervention and were sacrificed at 3 h, 6 h and 24 h after weaning of CPB respectively (*n* = 5 independent experiments). (B) Representative haematoxylin and eosin staining images in Sham group, DHCA 3 h group, DHCA 6 h group and DHCA 24 h group. The DHCA 3 h group rats showed prominent alveolar inflammatory cells infiltration, mainly comprising of mononuclear cells and granulocytes. The DHCA 6 h group exhibited interstitial capillary vessel dilatation and congestion, alveolar haemorrhage and interstitial oedema (Scale bar = 100 μm). (C) Histologic analysis for lung injury. The total histopathological score was expressed as the sum of the scores for all parameters. (D) Compared with the Sham group, alveolar total protein concentration significantly increased. (E) The lung wet/dry weight ratio also increased, although it did not reach a statistical difference. (F) The levels of inflammatory factors in bronchoalveolar lavage fluid (BALF) and blood serum were significantly increased when compared with the Sham group. (G) Ultrastructural changes were observed by electron microscopy. In the Sham group, type II alveolar epithelial were attached to intact and continuous basement membranes, with coarse and short microvilli present on the free surface of the cells, intermediate nuclei, abundant cytoplasm and normal mitochondrial structure, characterized by fingerprint‐like or concentric circle‐like lamellar microsomes. In contrast, type II alveolar epithelial from the DHCA 6 h group display relatively electron‐lucent nuclei and cytoplasm, reduced microvilli, fine vacuolar‐like changes in the cytoplasm and increased lysosomes. In addition, mitochondrial swelling and hydropic change were observed (Scale bar = 2 μm). *p < 0.05; **p < 0.01; ***p < 0.001

A total of 24 rats were randomly assigned to Protocol I (S group, *n* = 5; D3 h group, *n* = 5; D6 h group, *n* = 5; D24 h group, *n* = 5, 4 rats that received DHCA intervention died) to assess lung injury secondary to DHCA. For the 19 rats subject to DHCA intervention, 15 rats survived. In contrast, 2 rats (1 from the D3 h group and the other from the D24 h group) experienced haemodynamic collapse at approximately 5 h after surgery, and an additional 2 rats from the D6 h group experienced sudden death after recovery from anaesthesia due to respiratory failure, which was validated by autopsy. All S group rats successfully completed the sham operation. The haemodynamic parameter data obtained from each group of rats (Table 1) demonstrated no differences in MAP between the S, D3 h, D6 h and D24 h groups; however, blood gas analysis showed significant decreases in both Hb and Hct levels in DHCA‐treated rats compared with the S group rats. No differences were found between the different DHCA group rats (D3 h, D6 h, and D24 h groups), which was due to blood dilution with the blood priming process during CPB execution. PaO_2_ was not altered based on the DHCA procedure, and lactic acid accumulation occurred with the completion of a 50‐min circulation arrest for which sodium hydrogen carbonate was deliberately administered to correct acidosis. These data indicate that our modified CPB‐aided DHCA procedure was successfully delivered to most of the rats with reasonable results obtained in *Protocol I* (Figure 2A).

**TABLE 1:** Mean arterial pressure and blood gas analyses during deep hypothermic circulatory arrest

Group	MAP (mmHg)	Hb (g/dL)	Hct (%)	PaO_2_ (mmHg)	PaCO_2_ (mmHg)	pH	Lac (mmol/L)
Sham							
T1	129.8 ± 19.6	142.0 ± 4.0	43.5 ± 1.3	244.3 ± 71.0	39.4 ± 1.4	7.4 ± 0.0	1.5 ± 0.7
T5	–	147.0 ± 1.7	44.7 ± 1.0	181.7 ± 69.9	35.1 ± 2.0	7.4 ± 0.0	1.7 ± 0.3
DHCA 3 h							
T1	110.7 ± 3.5	151.0 ± 12.3	46.3 ± 3.8	124.0 ± 21.0	34.9 ± 6.9	7.5 ± 0.1	3.7 ± 1.9
T2	108.4 ± 17.7	95.3 ± 6.5	29.2 ± 2.0	411.0 ± 111.0	34.6 ± 8.3	7.5 ± 0.1	2.0 ± 0.7
T3	118.4 ± 14.4	102.0 ± 7.5	31.3 ± 2.2	302.3 ± 61.0	29.5 ± 7.7	7.5 ± 0.1	8.8 ± 1.0
T4	103.3 ± 19.5	102.3 ± 9.5	31.3 ± 2.9	305.0 ± 33.0	27.0 ± 16.1	7.5 ± 0.2	7.1 ± 2.5
T5	–	113.0 ± 10.8	34.6 ± 3.3	134.7 ± 15.6	26.8 ± 1.3	7.5 ± 0.0	4.7 ± 1.3
DHCA 6 h							
T1	120.2 ± 7.5	138.7 ± 11.8	42.7 ± 3.6	169.7 ± 4.9	33.8 ± 16.2	7.5 ± 0.1	3.1 ± 0.4
T2	108.6 ± 11.4	84.7 ± 13.2	25.9 ± 4.1	352.7 ± 100.4	38.8 ± 7.7	7.4 ± 0.1	1.3 ± 0.1
T3	120.1 ± 11.3	91.3 ± 7.8	28.0 ± 2.5	367.7 ± 133.4	34.2 ± 4.3	7.4 ± 0.1	7.9 ± 1.7
T4	122.0 ± 7.9	101.3 ± 22.0	31.1 ± 6.5	166.4 ± 86.1	24.7 ± 3.8	7.5 ± 0.1	8.4 ± 0.8
T5	–	82.7 ± 14.3	25.3 ± 4.5	92.2 ± 13.4	42.1 ± 4.0	7.4 ± 0.0	3.2 ± 1.2
DHCA 24 h							
T1	124.5 ± 9.1	133.5 ± 32.4	41.0 ± 10.1	150.2 ± 70.8	32.4 ± 10.4	7.5 ± 0.1	3.0 ± 1.7
T2	127.5 ± 10.5	101.0 ± 7.1	31.2 ± 1.8	342.5 ± 3.5	26.6 ± 5.0	7.6 ± 0.1	2.0 ± 0.5
T3	123.2 ± 22.0	96.7 ± 15.9	29.7 ± 4.7	266.3 ± 40.1	35.9 ± 14.0	7.5 ± 0.0	8.9 ± 2.3
T4	119.2 ± 5.8	108.7 ± 8.1	33.1 ± 2.1	292.3 ± 66.3	28.1 ± 12.8	7.5 ± 0.2	7.9 ± 1.6
T5	–	98.0 ± 5.3	29.9 ± 1.4	125.3 ± 11.0	35.6 ± 8.2	7.5 ± 0.1	3.6 ± 0.7

Abbreviations: MAP, mean arterial pressure; Hb, haemoglobin; Hct, red blood cell specific volume; PaO_2_, partial pressure of arterial oxygen; PaCO_2_, partial pressure of arterial carbon dioxide; Lac, lactic acid; DHCA, deep hypothermic circulatory arrest; DHCA 3 h, 3 hours post of DHCA; T1, 10 minutes pre‐CPB; T2, precardiac arrest; T3, cardiac rebeating; T4, weaning of CPB; T5, before been sacrificed.

DHCA‐exposed rats exhibited a dynamic phase‐distinct phenotype of lung injury. D3 h group rats showed prominent alveolar inflammatory infiltration mainly involving mononuclear cells and granulocytes (Figure 2B, C), whereas the D6 h group exhibited interstitial capillary vessel dilatation and congestion, alveolar haemorrhage and interstitial oedema, indicating an impaired microvascular barrier (Figure 2B, C). Lung tissue injury secondary to DHCA was further confirmed by the increases in BALF protein (Figure 2D), wet/dry weight ratio (Figure 2E) and levels of inflammatory cytokines (Figure 2F). Transmission electron microscopy displayed pathological changes in alveolar ultrastructure in the D6 h group rats. These changes were characterized by increased electron transparency of the nucleus and cytoplasm and mitochondrial swelling and widening of cristae junctions. These changes were especially prominent in type II alveolar epithelial cells (Figure 2G). Of note, these structural changes partially recovered at 24 h post DHCA, indicating that lung injury mainly occurs approximately 3–6 h after completion of the DHCA procedure, which also explains why sudden death mainly occurred within approximately 6 h after DHCA in our present study.

### 3.2 Dynamic changes in autophagic activities following DHCA

**FIGURE 3:**
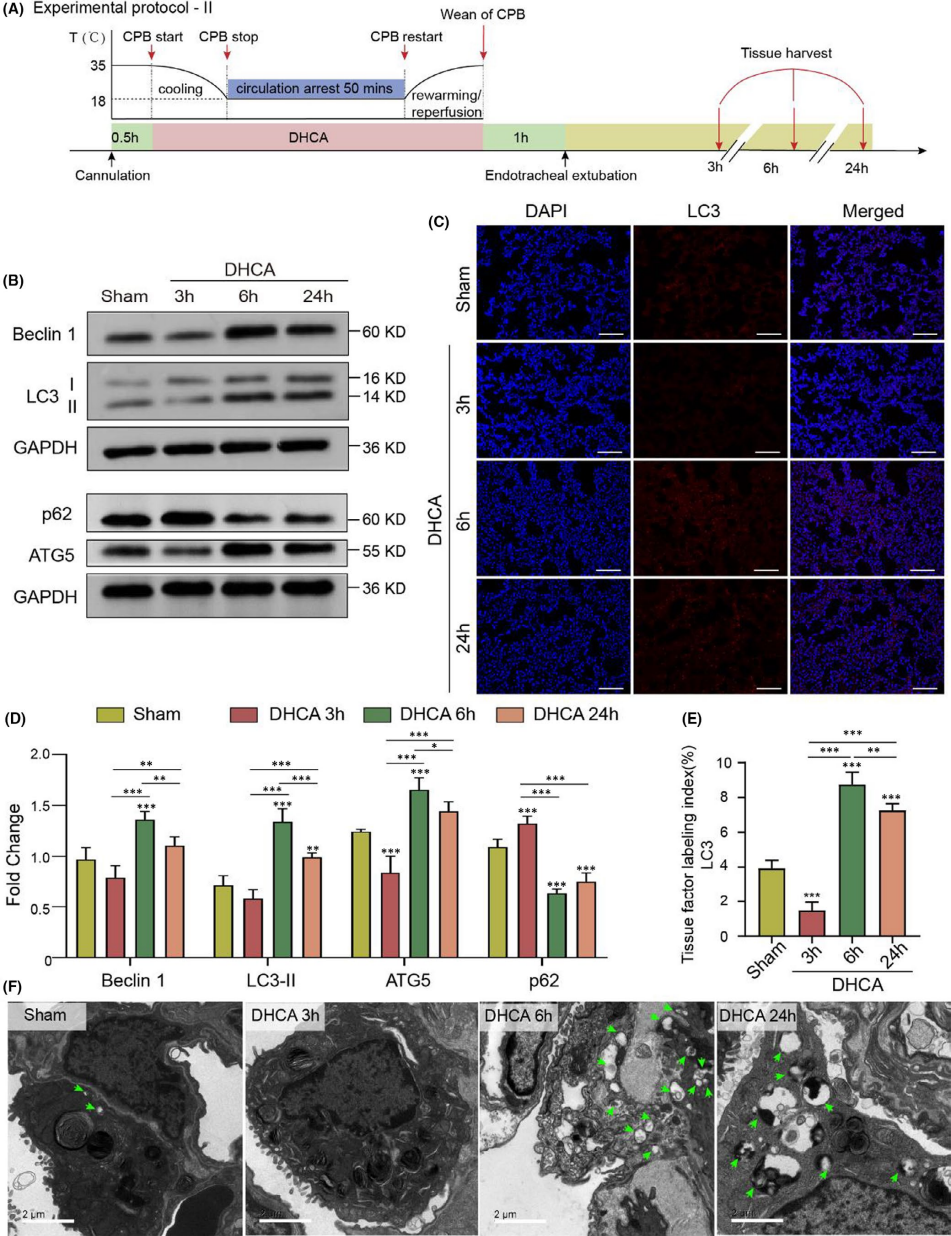
Dynamic Changes in Autophagy Activities Following DHCA. (A) Experimental Protocol II (see Experimental Protocol I for details). (B and D) The expression levels of LC3‐II, Beclin 1, ATG5 were slightly lower in DHCA 3 h group rats. However, the expression of autophagy‐related proteins in DHCA 6 h group was gradually recovered, which was eventually even higher than those in Sham group rats, and surprisingly augmented autophagy activity showed normalization process at 24 h after DHCA. Quantitative analysis of autophagy‐related proteins is shown in (D). Expression levels of autophagy‐related proteins were quantified, and their absolute changes were calculated and analysed by one‐way ANOVA shown in (D; *n* = 5). (C and E) Compared to the Sham group, immunofluorescence staining for LC3‐II showed a slight decrease in the number of puncta in the DHCA 3 h group, a significant increase in the number of puncta in the DHCA 6 h and DHCA 24 h groups (Scale bar = 100 μm). Red, LC3‐positive cells; blue, DAPI‐stained nuclei. Quantitative analysis is shown in (E; *n* = 5). (F) The formation and number of autophagosomes and autophagolysosomes were further observed by electron microscopy, and the results were consistent with the western blotting findings (Scale bar = 2 μm)

A set of autophagy machinery proteins, including LC3‐II, Beclin 1, ATG5, and p62,^28^ was carefully measured using lung tissue obtained from rats in different designated groups to assess autophagy activity. The dynamic changes in autophagy activity were quantified according to Protocol II (Figure 3A). Compared with the S group, the expression levels of LC3‐II, Beclin 1, and ATG5 were slightly lower in the D3 h group rats (Figure 3B, D, full blots are shown in Figure S1A, private link: https://figshare.com/s/9d775fb577d553c5e7c5, DOI: https://doi.org/10.6084/m9.figshare.14938407.v1), indicating that autophagy was suppressed at the early phase after DHCA. In contrast, at the late stage (at 6 h) after DHCA, the D6 h group rats showed a gradual recovery of autophagy‐related protein expression (Figure 3B, D), which was eventually even higher than that in the S group rats. Surprisingly, altered autophagic activity induced by DHCA eventually returned to a similar level as that in the S group 24 h after DHCA (Figure 3B, D).

Consistent with Western blot findings, the immunofluorescence staining showed a decrease in LC3‐II expression level at the early stage (3 h) after DHCA compared with S group rats; however, the level was even higher than that of S group rats at the late stage (6 h) (Figure 3C, E) after DHCA. Transmission electron microscopy examination further confirmed this pattern of changes in autophagy activity at the two specific time points after the DHCA procedure (Figure 3F).

**FIGURE 4:**
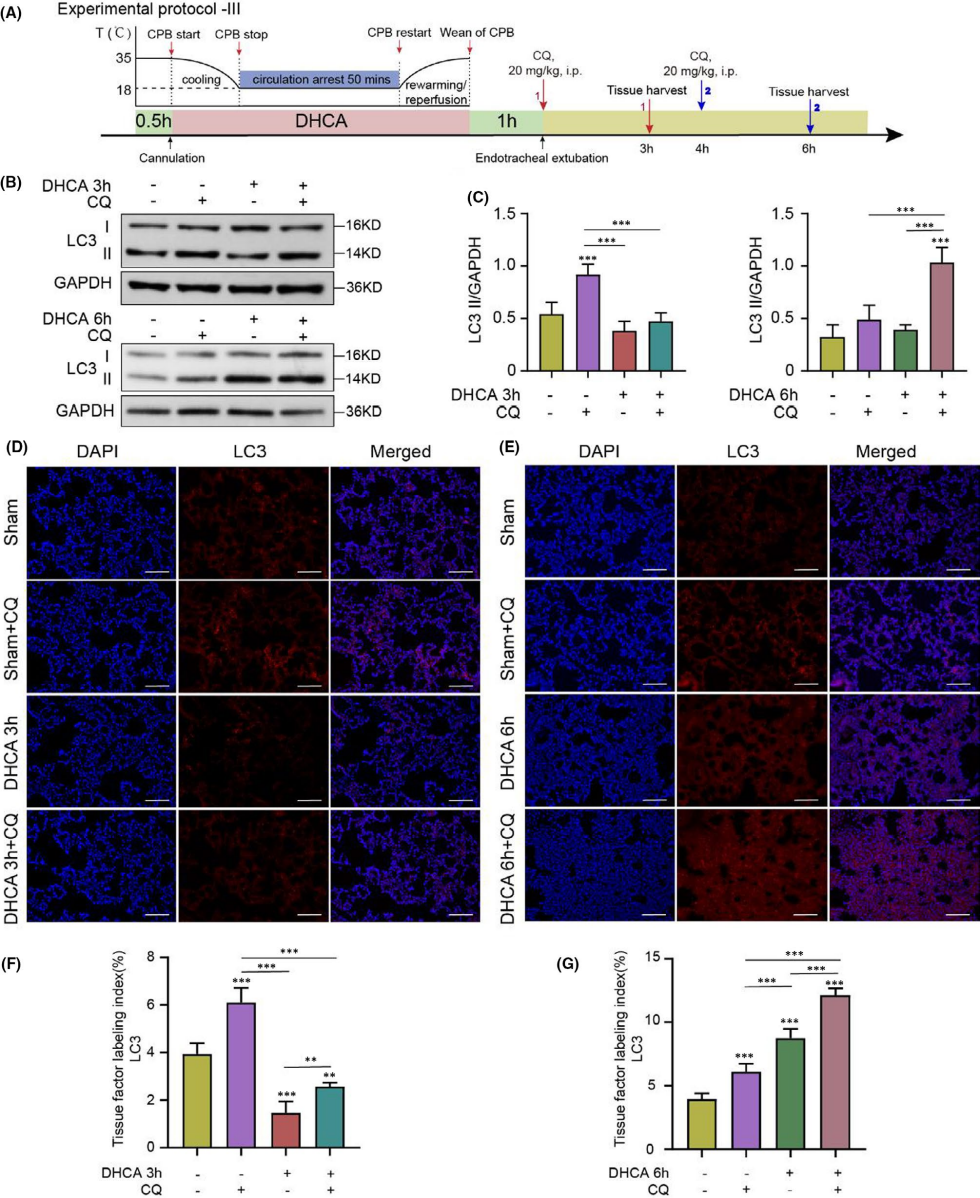
Chloroquine Was Used to Determine Whether Interrupted Autophagic Flux Contributed to the Increase in LC3‐II Level. (A) Rationale and design of Experimental Protocol III. The Sham, DHCA 3 h, and DHCA 6 h group rats were given chloroquine (CQ) 2 h before euthanization (*n* = 3 independent experiments). (B and C) The expression of LC3‐II increased in Sham + CQ group, DHCA 3 h + CQ group and DHCA 6 h + CQ group compared to their respective Control group. Quantitative analysis is shown in (C; *n* = 5). (D and F) Compared to DHCA 3 h group, immunofluorescence staining for LC3‐II showed a significant increase in the number of puncta in the DHCA 3 h + CQ group (Scale bar = 100 μm). Red, LC3‐positive cells; blue, DAPI‐stained nuclei. Quantitative analysis is shown in (F; *n* = 5). (E and G) Compared to DHCA 6 h group, immunofluorescence staining for LC3‐II showed a significant increase in the number of puncta in the DHCA 6 h + CQ group (Scale bar = 100 μm). Red, LC3‐positive cells; blue, DAPI‐stained nuclei. Quantitative analysis is shown in (G; *n* = 5)

To further evaluate the dynamic changes in autophagy activity in the lung tissue following the DHCA procedure, CQ was delivered to rats in both the D3 h and D6 h groups to measure autophagic flux (see *Protocol III* for details, Figure 4A). Interestingly, we observed that CQ intervention resulted in increases in LC3‐II levels in both D3 h and D6 h group rats compared with rats without CQ (Figure 4B–G, whole blots are shown in Figure S1B, private link: https://figshare.com/s/9d775fb577d553c5e7c5, DOI: https://doi.org/10.6084/m9.figshare.14938407.v1), indicating that DHCA itself did not interrupt the autolysosomal degradation process.

Taken together, our results suggested dynamic changes in autophagy activities in the lung tissue at the early and late phases following DHCA, whereas autophagic flux was not interrupted following DHCA.

### 3.3 Autophagy activation at the early phase of DHCA aggravated lung injury

**FIGURE 5:**
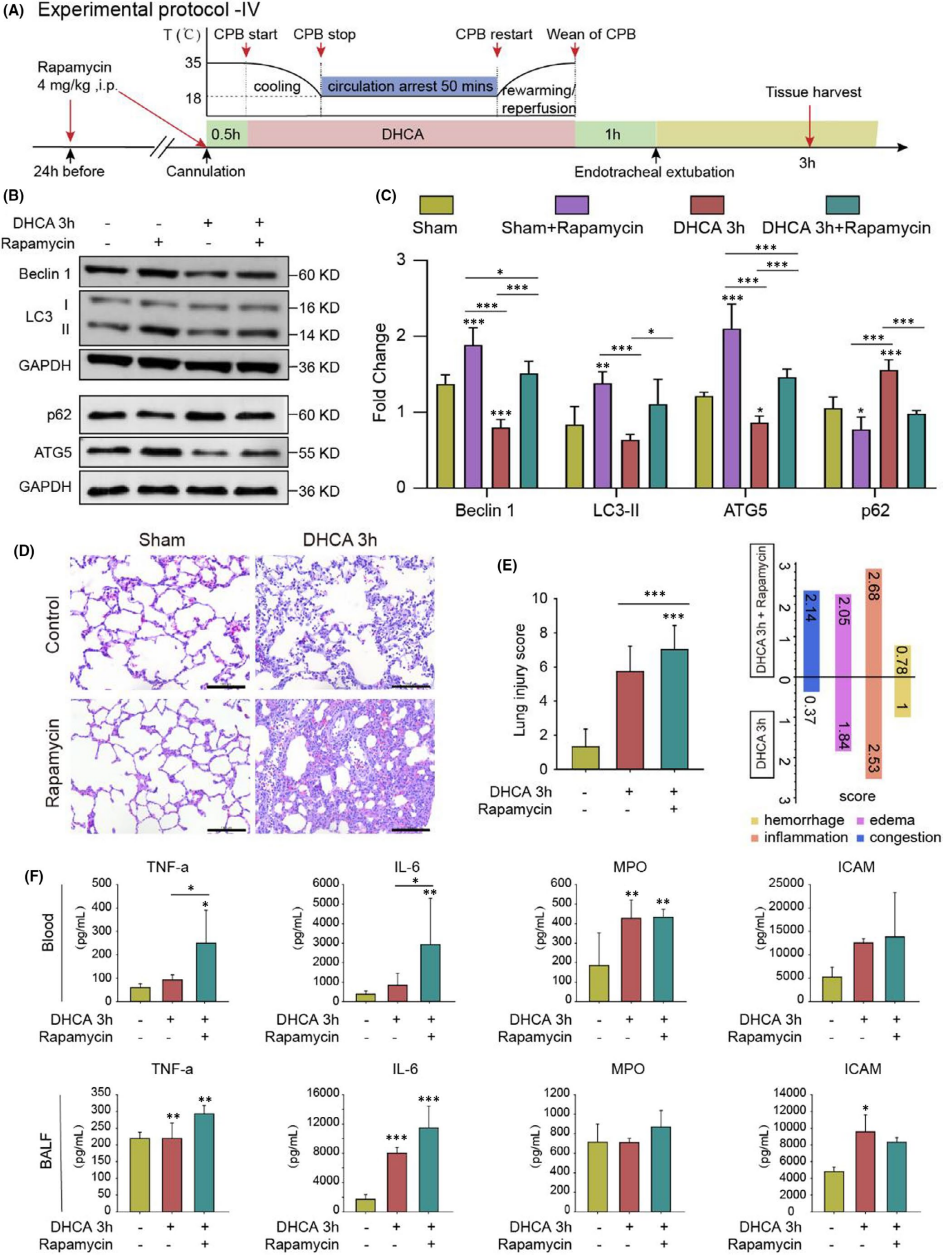
Re‐activation of suppressed autophagy at the early phase of DHCA aggravated lung injury. (A) Experimental Protocol IV. Autophagy inducer rapamycin was given by intraperitoneal injection at 24 h, 30 min pre‐operation in Sham group and DHCA 3 h group, respectively (*n* = 5 independent experiments). (B and C) The expression levels of autophagy‐related proteins were significantly increased after rapamycin was given. Quantitative analysis is shown in (C; *n* = 5). (D) Representative haematoxylin and eosin staining images in Sham group (as Control), DHCA 3 h group (as Control), Sham +rapamycin group and DHCA 3 h + rapamycin group. Compared with the Control group, the degree of lung injury increased at 3 h after DHCA (Scale bar = 100 μm). (E) Re‐activation of autophagy significantly increased lung injury score at the early phase of DHCA. (F) The levels of inflammatory factors in blood serum and BALF were significantly high after the re‐activation of autophagy. *p < 0.05; **p < 0.01; ***p < 0.001

Rapamycin, a typical autophagy inducer, was delivered at 24 h and 30 min pre‐operation (i.p.) to re‐activate the suppressed autophagy activity at the early phase (3 h) after DHCA (see Protocol IV for details, Figure 5A). Rapamycin increased autophagic protein expression levels in D3 h group rats, which reached similar levels as S group rats (Figure 5B, C, whole blots on PVDF membrane shown in Figure S1C, private link: https://figshare.com/s/9d775fb577d553c5e7c5, DOI: https://doi.org/10.6084/m9.figshare.14938407.v1). Unexpectedly, rapamycin augmented lung injury, as evidenced by worsened inflammatory cell infiltration, pulmonary oedema and alveolar septal congestion shown in both histology examinations than in D3 h group rats treated with placebo therapy (Figure 5D, E). Moreover, quantifications for both BALF and serum cytokines showed similar results (Figure 5F). Thus, our data indicated that suppressed autophagy is probably an adaptive response to changes in microenvironments at the early phase after DHCA.

### 3.4 Inhibition of autophagy at the late stage of DHCA alleviated pulmonary injury

**FIGURE 6:**
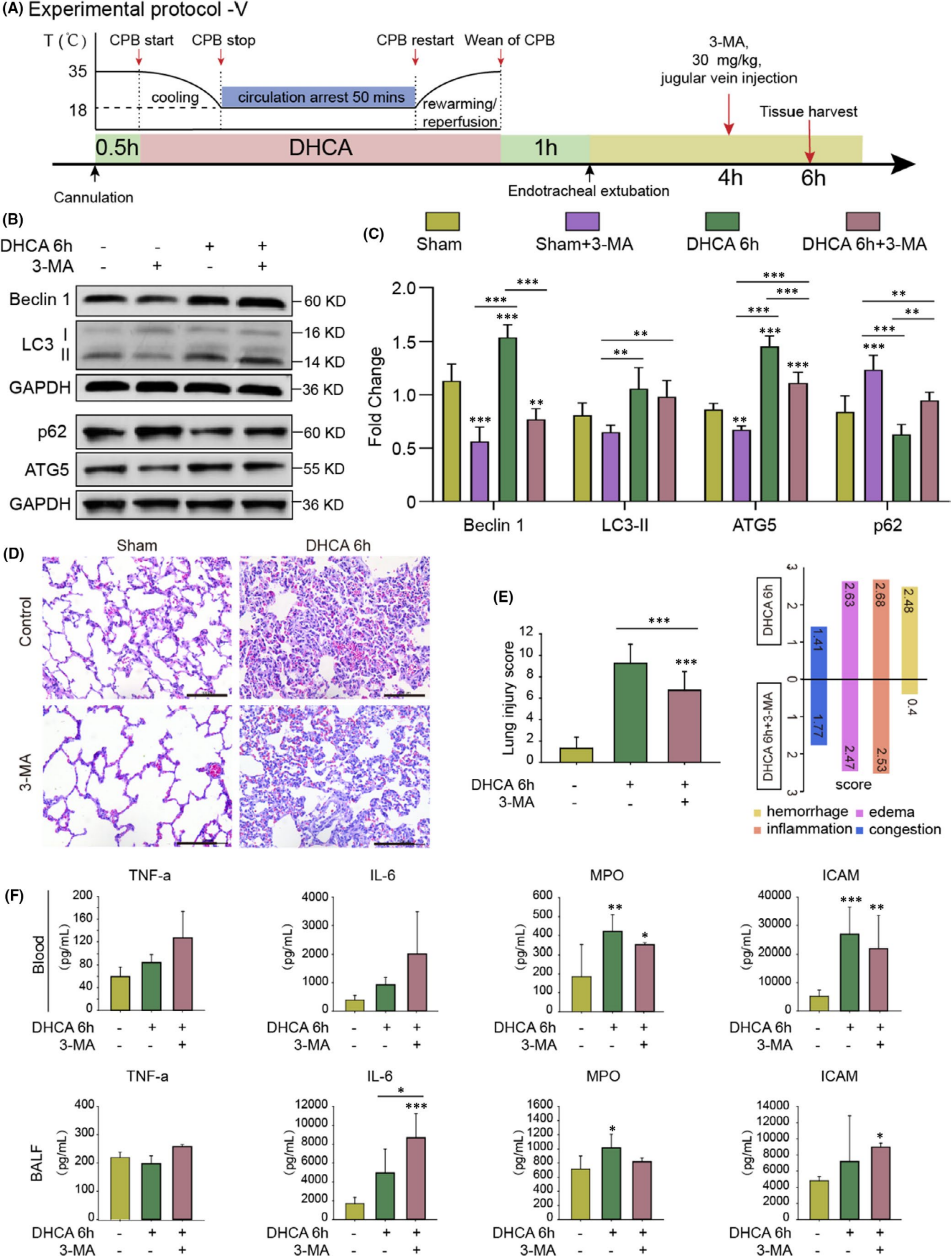
Inhibition of Autophagy at the Late Stage of DHCA Alleviated Pulmonary Injury. (A) Experimental Protocol V. Autophagy inhibitor 3‐methyladenine (3‐MA) was given by intraperitoneal injection at 2 h before euthanization in Sham group and DHCA 6 h group (*n* = 3 independent experiments). (B and C) The expression levels of autophagy‐related proteins were significantly decreased after 3‐MA was given. Quantitative analysis is shown in (C; *n* = 5). (D) Representative haematoxylin and eosin staining images in Sham group (as Control), DHCA 6 h group (as Control), Sham + 3‐MA group and DHCA 6 h + 3‐MA group. Compared with the DHCA 6 h group, the degree of lung injury in DHCA 6 h + 3‐MA group decreased (Scale bar = 100 μm). (E) Inhibition of autophagy significantly decreased lung injury score at the late stage of DHCA. (F) The levels of some inflammatory factors in blood serum and BALF showed a trend of decreases, albeit not statistically significant, after the inhibition of autophagy. *p < 0.05; **p < 0.01; ***p < 0.001

Given that increased autophagic activity was observed at a late phase after (6 h post) DHCA, 3‐MA, an autophagy inhibitor, was then administered 2 h before euthanization (jugular vein injection) to rats to further investigate the role of autophagy in lung injury late after the DHCA procedure (Protocol V, Figure 6A). We noted that 3‐MA exposure resulted in decreased levels of LC3‐II, Beclin 1 and ATG5 in lung tissue at 6 h post DHCA (Figure 6B, C, full blots are shown in Figure S1D, private link: https://figshare.com/s/9d775fb577d553c5e7c5, DOI: https://doi.org/10.6084/m9.figshare.14938407.v1), which was associated with a reduced extent of lung injury, especially alveolar haemorrhage (Figure 6D, E). Moreover, the improved lung injury was associated with a trend of, albeit not statistically significant, reductions in the levels of inflammatory factors in lung tissue (Figure 6F). Thus, these data indicated that overactivation of autophagy leads to pathological lung damage in the relatively late phase after DHCA.

## 4 DISCUSSION

In our study, we showed that a natural inflammatory response occurred in the lung tissue following the DHCA procedure, which caused a unique phase‐dependent phenotype of lung injury. Coincidently, the activity of autophagy after DHCA was decreased at the early phase (3 h following DHCA procedure) but was increased at the late phase (6 h after). Interestingly, autophagic flux was not interrupted at either the early or late phase following DHCA, as CQ delivery caused further increases in the expression levels of autophagy machinery proteins. Importantly, when rapamycin was administered at the early phase after DHCA to augment autophagic activity, it caused a worsening of lung injury. These results indicate that suppressed autophagy at the early phase following DHCA is probably an adaptive response that plays a protective role. In contrast, 3‐MA delivered at the late stage following DHCA to inhibit excessive activated autophagy attenuated lung injury, indicating that augmented autophagy is responsible for the pathological injury induced by DHCA. Thus, our study is the first to provide detailed information on the dynamic inflammatory response following DHCA and the time dependent role of autophagy in DHCA‐induced lung injury.

A large body of data indicate that autophagy plays key roles not only in maintaining cellular homeostasis but also in the pathological process of many diseases,^29^ including neurodegenerative diseases,^30^ cardiovascular diseases,^31^ age‐related diseases^32^ and pulmonary diseases.^11^, ^33^ Studies have shown that autophagy plays a very complex role in the pathogenesis of lung injury that is either protective or detrimental roles.^11^, ^17^, ^34^ Although, to date, very few studies have investigated the role of autophagy in ALI induced by I/R. It has been shown that autophagy plays a protective role in I/R‐induced lung injury^14^; accordingly, inhibition of autophagy aggravates lung injury.^16^ On the other hand, overactivation of autophagy can cause further damage to the lung tissue. A study has shown that upregulated autophagy exacerbates lung I/R injury.^7^ The different data obtained from these studies could be due to the different extents of ischaemia (ie the infarct area) or different experimental setups that exist in different research laboratories. Indeed, the different data actually indicate that a moderately functioning autophagy process is essential for maintaining normal homeostasis in lung tissue. In the present study, our data suggest that autophagy in general contributes to DHCA‐induced lung injury. In the early phase after DHCA, a reversal of autophagy activity by rapamycin resulted in augmented lung injury. However, in the late phase following DHCA, suppression of autophagy activity attenuated lung injury. Thus, our data strongly suggest that in our specific rat model of DHCA‐induced lung injury, autophagy has a detrimental effect on lung injury rather than a protective effect.

### 4.1 Study limitations and future perspectives

Several limitations in our study should be emphasized. The normal lung is dynamic heterogeneous tissue comprising strikingly different types of cells exhibiting their own unique biological characteristics. Therefore, one would assume the autophagy activity within the lung tissue will be very dynamic and would exhibit a wide spectrum of autophagy reactions in terms of activity in response to DHCA insult. Moderate appropriate autophagy will be highly desirable to maintain lung tissue homeostasis. Although the induction of autophagy activation by rapamycin results in a worsening of lung injury, we failed to test different dose effects on the role of autophagy activation in DHCA‐induced lung injury due to the difficulty of animal model setup. It might well be that a lower dose of rapamycin for which autophagy is only slightly, moderately elevated, would even show protective effects on lung tissue against injury at the early phase after DHCA. In addition, in the clinical scenario, patients who undergo cardiac surgery generally have comorbidities, such as diabetes, hypertension and other systemic organ dysfunction; therefore, our data might not be fully translated into clinical applications. A sophisticated experimental design in which the DHCA model is implemented in diabetic or hypertensive animals is needed for better translational purposes. Furthermore, it must be kept in mind that DHCA induces systemic inflammatory responses,^35^ which can cause multiple system organ dysfunctions directly, such as brain^36^ or gut^35^ injury. Therefore, systemic inflammatory responses can trigger interorgan cross talk, which has been observed in intestinal I/R preconditioning‐mediated lung protection against prolonged I/R injury^37^ possibly through an improved innate immune response.^38^ Finally, although we provided a unique set of data demonstrating the time‐dependent effects of autophagy on the pathological process after DHCA, which aimed to help us better understand the complicated roles of autophagy, the detailed underlying mechanisms remain largely unknown. Specifically, how autophagy is regulated, especially how it is suppressed and then augmented at the early versus the late phase after DHCA, remains unclear. Many other factors, such as temperature control and the CPB material used, should also be carefully taken into account in our DHCA‐mediated lung injury model.^39^ Nevertheless, deep insights into this differential regulatory mechanism would reveal unique molecular markers that will guide our therapeutic treatment for better clinical outcomes.

In conclusion, DHCA can induce a time‐dependent pathological process in lung tissue, for which a dynamic change in autophagy activity characterized by suppressed autophagy at the early phase followed by considerably augmented autophagy at the late phase after DHCA plays an important role. Importantly, induction of autophagy activation by rapamycin at the early phase actually worsened lung injury, suggesting an adaptive decrease in autophagy. In contrast, 3‐MA‐mediated suppression of autophagy attenuates lung injury at the late phase after DHCA. These data suggest that DHCA‐mediated autophagic activation contributes to the lung injury observed in our present study. Further studies are needed to elucidate the underlying mechanisms of how autophagy is regulated following the DHCA procedure.

## CONFLICTS OF INTEREST

No conflicts of interest, financial or otherwise, are declared by the authors.

## AUTHOR CONTRIBUTIONS

**Minjian Kong:** Conceptualization (equal); Resources (equal); Software (equal); Supervision (equal); Validation (equal); Writing – original draft (equal); Writing – review & editing (equal). **Dongdong Wei:** Conceptualization (equal); Resources (equal); Writing – original draft (lead); Writing – review & editing (equal). **Xuebiao Li:** Conceptualization (equal); Formal analysis (equal); Project administration (equal); Resources (equal); Software (equal); Writing – original draft (equal). **Xian Zhu:** Conceptualization (equal); Methodology (equal); Resources (equal); Validation (equal). **Ze Hong:** Formal analysis (equal); Resources (equal). **Ming Ni:** Conceptualization (equal); Data curation (equal); Resources (equal); Software (equal). **Yifan Wang:** Conceptualization (equal); Data curation (equal); Resources (equal); Software (equal). **Aiqiang Dong:** Conceptualization (equal); Data curation (equal); Formal analysis (equal); Resources (lead); Supervision (lead); Validation (lead); Writing – review & editing (equal).

## PATENTS

The patent for the cardiac pulmonary bypass device was published by the State Intellectual Property Office, the application number, 201910261956X.

## Supporting information

Appendix S1
