# Supplementary material for: The dynamic changes in autophagy activity and its role in lung injury after deep hypothermic circulatory arrest
Source: J Cell Mol Med. 2022 Jan 11;26(4):1113–27. doi: 10.1111/jcmm.17165 (PMC8831962; doi:10.1111/jcmm.17165)
Supplement: Supplementary file 1 — Appendix S1 [file JCMM-26-1113-s001.docx]

**Supplemental Material**

**Supplemental Methods**

***Cell Culture***

The human bronchial epithelioid cell line 16HBE was cultured in Dulbecco’s Modified Eagle’s Medium (DMEM, Gibco) supplemented with 10% fetal bovine serum (FBS, Hyclone, Shanghai, China) and 1% penicillin/streptomycin (Gibco), in humidified air at 37°C with 5% CO_2_.

***16HBE in Vitro Oxygen Glucose Deprivation (OGD)/Re-oxygenation (OGD/R) Model***

To mimic pulmonary I/R in vitro, the OGD/R procedure was performed as described previously(1, 2). Briefly, 16HBE cells were seeded into 96-well plates at a density of 1 × 10^4^ cells per well and cultured for 24 h. And then cells were washed twice with phosphate-buffered saline (PBS, pH 7.4) and then refreshed with the glucose-free DMEM (100 μl, Gibco). Cells were initially placed in hypoxic workstations (Whitley H35 Hypoxystation), equilibrated for 15 min with a continuous flux of gas (94.9% N_2_ / 5% CO_2_ / 0.1% O_2_). After that, cells subjected to OGD were incubated in the solution at 37°C for 6 h to produce oxygen deprivation. Experimental parameters were assayed at 24 h following re-oxygenation (returned to the norm-oxygenated DMEM containing glucose).

***Co-culture of 16HBE Cells and Serum of Experimental Rats***

16HBE cells were seeded into 96-well plates at a density of 1 × 10^4^ cells per well and cultured for 24 hours. And then we added the serum of experimental rats (50 μl) to the well and cultured them for 24 hours. To better analyze the effect of animal serum on 16HBE cells, we co-cultured the cells subjected to OGD/R with animal serum for 24 hours. At the time of reoxygenation, we added animal serum (50 μl) to cells subjected to OGD/R while adding the norm-oxygenated DMEM containing glucose.

***LC3 Immunofluorescence Staining***

LC3 Immunofluorescence was performed according to the manufacturer’s instructions (4108, Cell Signaling Technology, Shanghai). The culture medium was removed, washed three times with PBS, and then fixed with ice-cold 100% methanol for 10 min at -20°C. Rinse three times in 1X PBS for 5 minutes each. Block and permeabilize specimen in Blocking Buffer (1X PBS / 5% normal serum / 0.3% Triton™ X-100) for 60 minutes and then Incubate with primary antibody overnight at 4°C. Rinse three times in 1X PBS for 5 minutes each. Incubate specimen in fluorochrome-conjugated secondary antibody for 1–2 hours at room temperature in dark. Finally, cells were washed and incubated with DAPI (S2110, Solarbio, Beijing) for 5 minutes. Acquire images immediately using a fluorescence microscope (IX51, Olympus, Japan).

***TUNEL (Terminal Deoxynucleotidyl Transferase dUTP Nick end Labeling) Assay***

The lung tissues obtained from group rats that were designated for H&E staining were fixed in 10% neutral formalin, paraffin-embedded, and sliced (3 microns). After deparaffinization, the sections were stained with TUNEL (C1090, Beyotime, Shanghai). Cell nuclei were co-stained with DAPI and visualized through a fluorescent microscope (Leica, Shanghai, China). The fluorescence density was assessed using *Image J* software *(Image J* 1.53c, National Institutes of Health, Bethesda, MD, USA).

**Supplemental Results**

The results from our observations in the rat DHCA model were corroborated in vitro by culturing 16HBEs with the serum of experimental rats (16HBEs + Serum) under OGD/R conditions. A positive control is 16HBEs subjected to OGD/R for 24 hours (Online Fig. IIA). Experimental animal serum changed the LC3-II level of 16HBEs. Compared with 16HBEs + Sham (16HBE cells treated with the serum of group Sham rats for 24 hours), immunofluorescence staining of 16HBEs + DHCA 3h showed that the expression level of LC3-II was reduced (Online Fig. IIB); however, the level of 16HBEs + DHCA 6h was significantly higher than that of 16HBEs + Sham (Online Fig. IIB & Online Fig. IIC). Rapamycin increased the level of LC3-II (Online Fig. III), on the contrary, 3-MA reduced 16HBEs LC3-II levels (Online Fig. VI). Furthermore, we used the serum of group rats which was treated with chloroquine (CQ) and 16HBEs to coculture for 24 hours to monitor the autophagic flux in 16HBEs. Consistent with the data obtained from the in vivo study, a marked increase in the number of puncta was observed in 16HBEs + (Sham + CQ) compared with 16HBEs + Sham (Online Fig. V A & Online Fig. VI A). The level of 16HBEs + (DHCA 3h + CQ) was higher than that of 16HBEs + DHCA 3h (Online Fig. V), similarly, the level of 16HBEs + (DHCA 6h + CQ) was also higher than that of 16HBEs + DHCA 6h (Online Fig. VI). Our in vitro cell culture experimental results have addressed some important issues, which further indicated that autophagy is indeed involved in our DHCA induced lung injury.

In addition, TUNEL assay was performed on lung tissue to assess the level of apoptosis. The average number of TUNEL stained cells in each group accounted for less than 1%, and there was no statistical difference between the groups (Online Fig. VII).

**Supplemental References**

1. **Zhao LP, Ji C, Lu PH, Li C, Xu B, and Gao H**. Oxygen glucose deprivation (OGD)/re-oxygenation-induced in vitro neuronal cell death involves mitochondrial cyclophilin-D/P53 signaling axis. *Neurochem Res* 38: 705-713, 2013.

2. **Xu S, Li Y, Chen JP, Li DZ, Jiang Q, Wu T, and Zhou XZ**. Oxygen glucose deprivation/re-oxygenation-induced neuronal cell death is associated with Lnc-D63785 m6A methylation and miR-422a accumulation. *Cell death & disease* 11: 816, 2020.

**Figure legends**

**
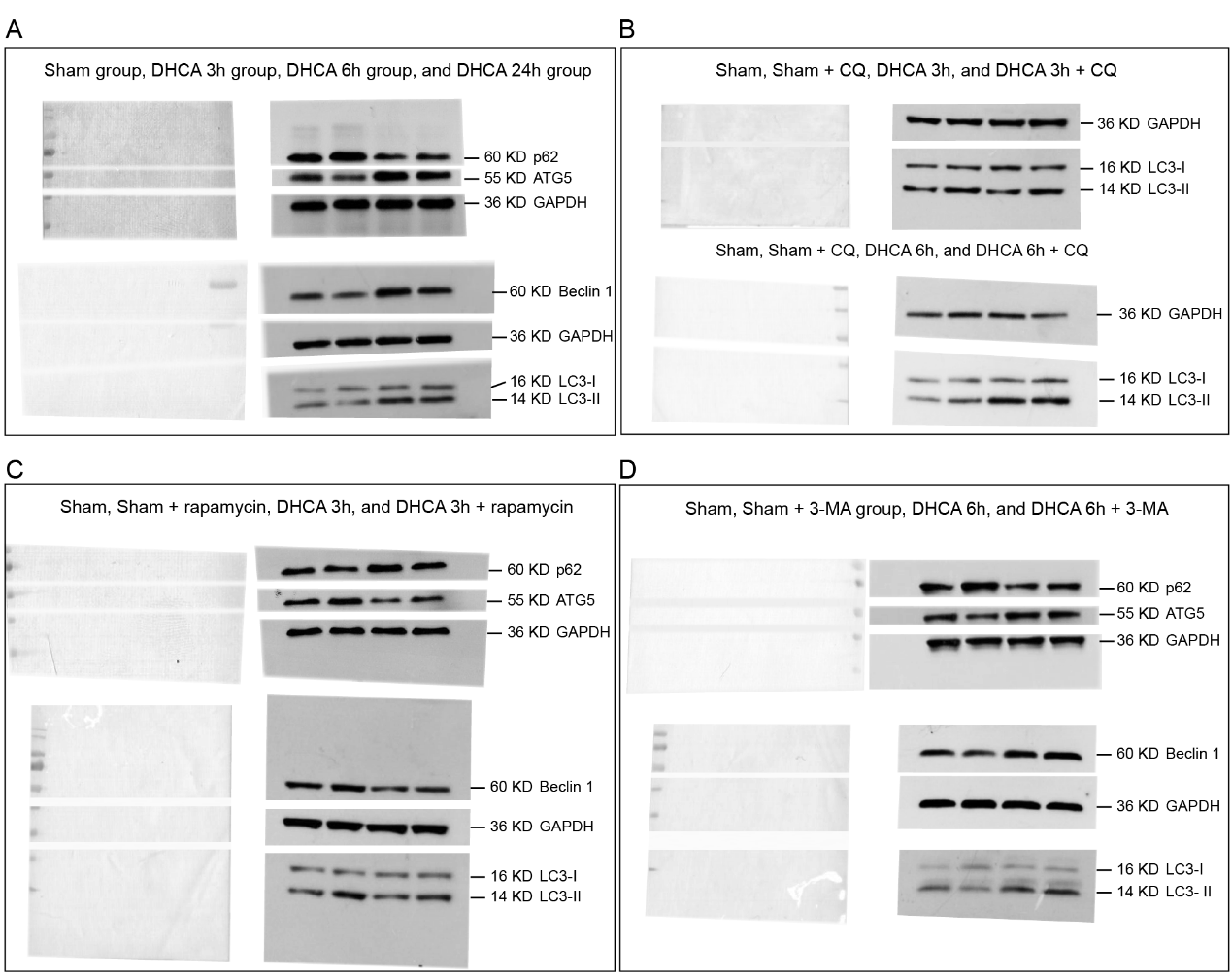
**

**Online Figure I.**

**Full-length gels and blots. (A)** A set of autophagy machinery proteins including LC3-II, Beclin 1, ATG5, and p62 has been measured using lung tissue obtained from different designated group rats (Sham group, DHCA 3h group, DHCA 6h group, and DHCA 24h group) to assess autophagy activity. **(B)** A set of autophagy machinery proteins including LC3-II, Beclin 1, ATG5, and p62 has been measured using lung tissue obtained from different designated group rats (Sham group, Sham + CQ group, DHCA 3h group, DHCA 3h + CQ group, DHCA 6h group, and DHCA 6h + CQ group) to assess autophagy activity. **(C)** A set of autophagy machinery proteins including LC3-II, Beclin 1, ATG5, and p62 has been measured using lung tissue obtained from different designated group rats (Sham group, Sham + rapamycin group, DHCA 3h group, and DHCA 3h + rapamycin group) to assess autophagy activity. **(D)** A set of autophagy machinery proteins including LC3-II, Beclin 1, ATG5, and p62 has been measured using lung tissue obtained from different designated group rats (Sham group, Sham + 3-MA group, DHCA 6h group, and DHCA 6h + 3-MA group) to assess autophagy activity.


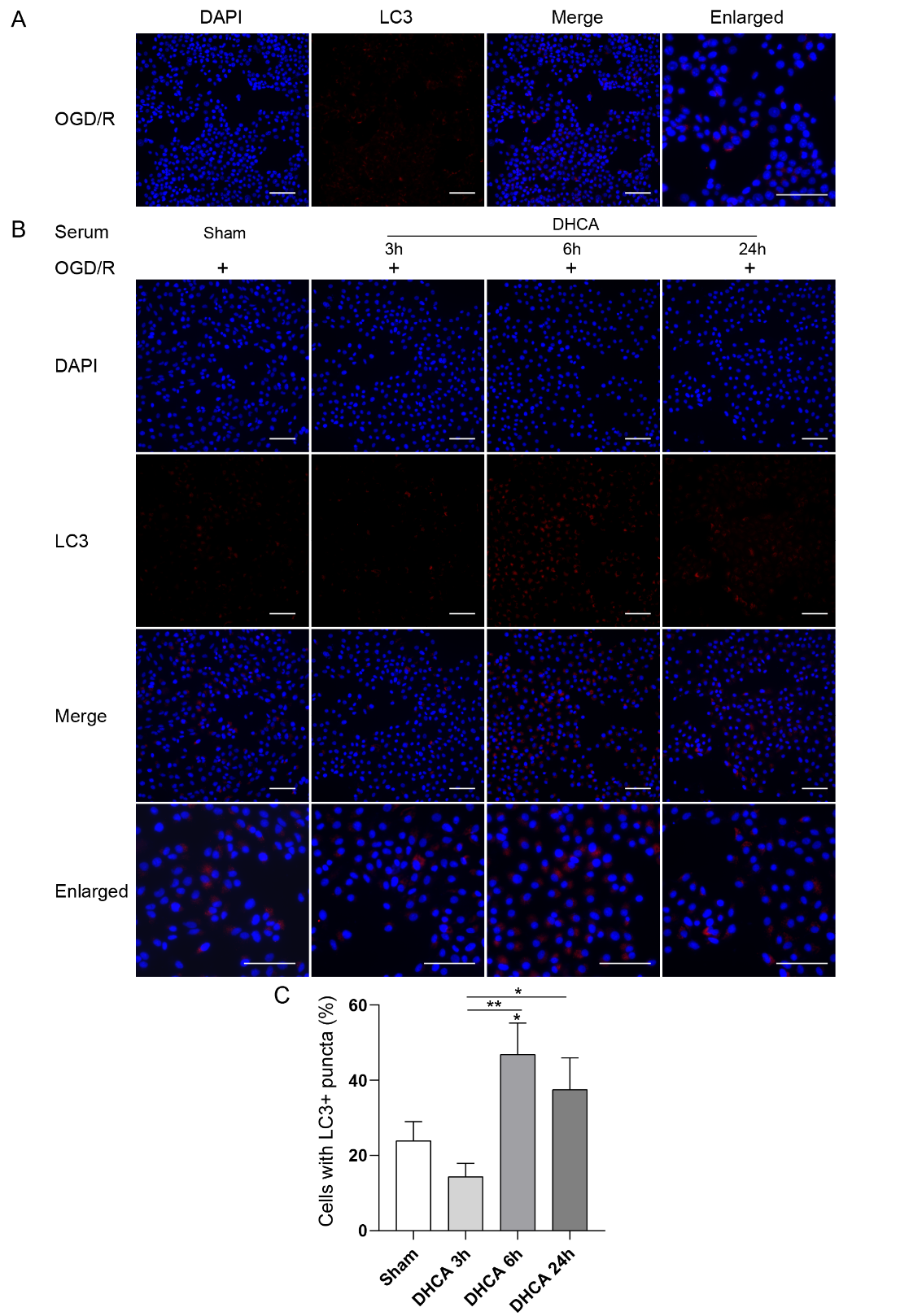


**Online Figure II. (A)** 16HBEs were subjected to OGD/R for 24 hours as a positive control. The enlarged picture is shown on the right. **(B)** Experimental animal serum changed the LC3-II level of 16HBEs. Compared with 16HBEs + Sham (16HBE cells treated with the serum of group Sham rats for 24 hours), immunofluorescence staining of 16HBEs + DHCA 3h showed that the expression level of LC3-II was reduced; however, the level of 16HBEs + DHCA 6h was significantly higher than that of 16HBEs + Sham. The enlarged picture is shown at the bottom (Bottom panel). (Scale bar=100 μm). Red, LC3-positive cells; blue, DAPI-stained nuclei. **(C)** Quantitative analysis.


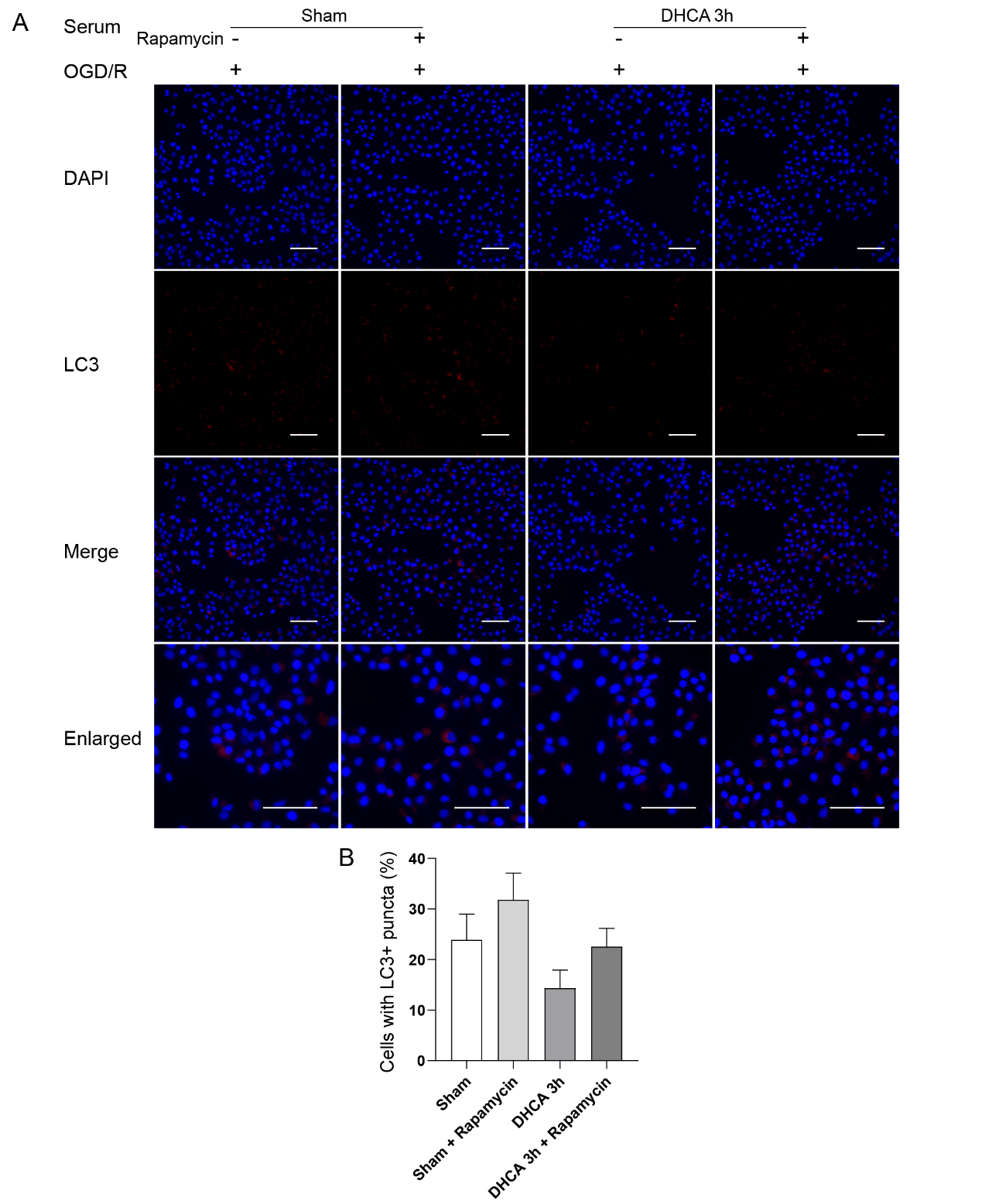


**Online Figure III. (A)** Compared with 16HBEs + Sham (16HBE cells treated with the serum of group Sham rats for 24 hours), immunofluorescence staining of 16HBEs + (Sham + rapamycin) showed that the expression level of LC3-II was increased; similarly, the level of 16HBEs + (DHCA 3h + rapamycin) was higher than that of 16HBEs + DHCA 3h. Rapamycin increased the level of LC3-II. The enlarged picture is shown at the bottom (Bottom panel). (Scale bar=100 μm). Red, LC3-positive cells; blue, DAPI-stained nuclei. **(B)** Quantitative analysis.


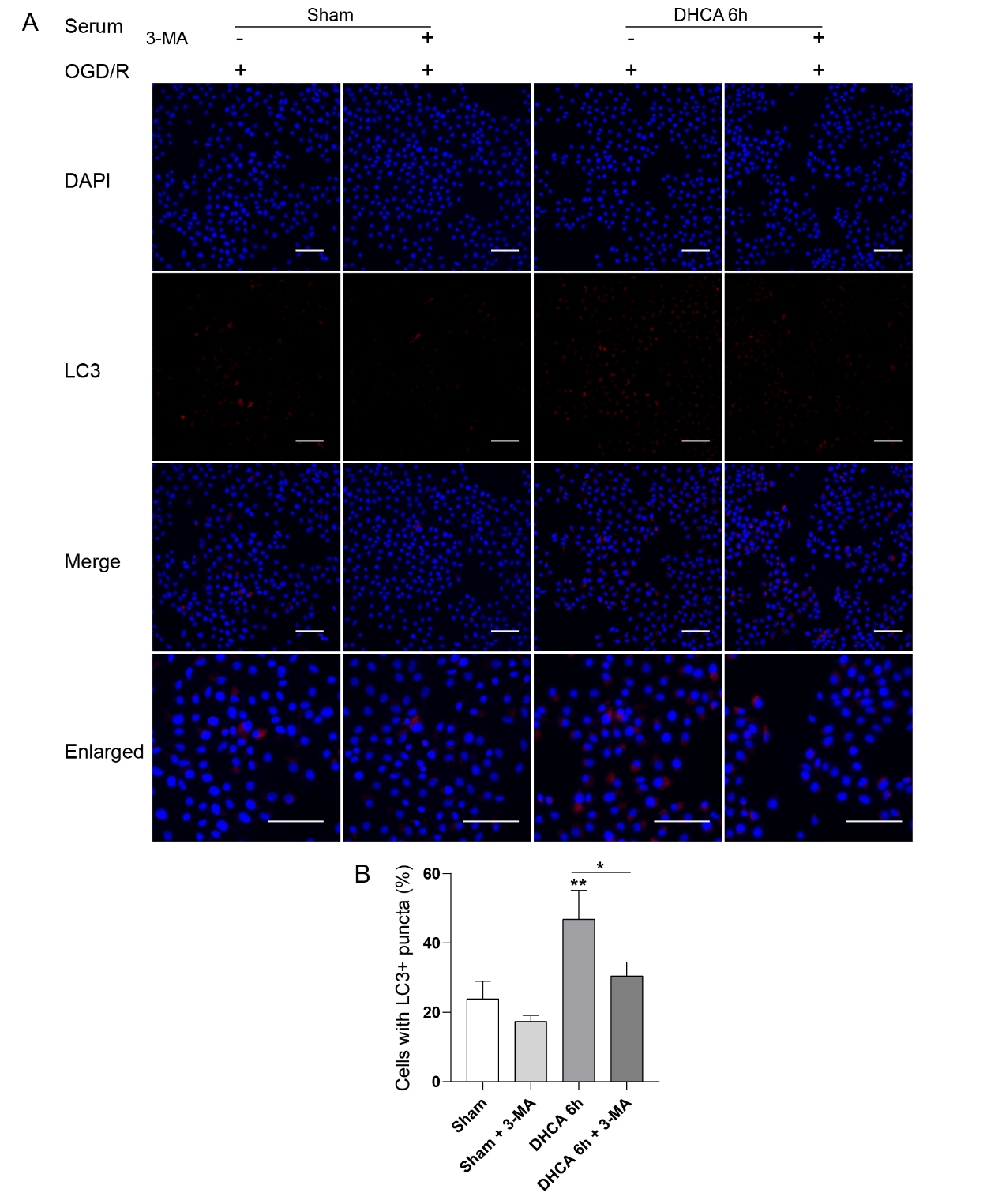


**Online Figure VI. (A)** Compared with 16HBEs + Sham (16HBE cells treated with the serum of group Sham rats for 24 hours), immunofluorescence staining of 16HBEs + (Sham + 3-MA) showed that the expression level of LC3-II was reduced; similarly, the level of 16HBEs + (DHCA 3h + 3-MA) was lower than that of 16HBEs + DHCA 3h. 3-MA reduced the level of LC3-II. The enlarged picture is shown at the bottom (Bottom panel). (Scale bar=100 μm). Red, LC3-positive cells; blue, DAPI-stained nuclei. **(B)** Quantitative analysis.


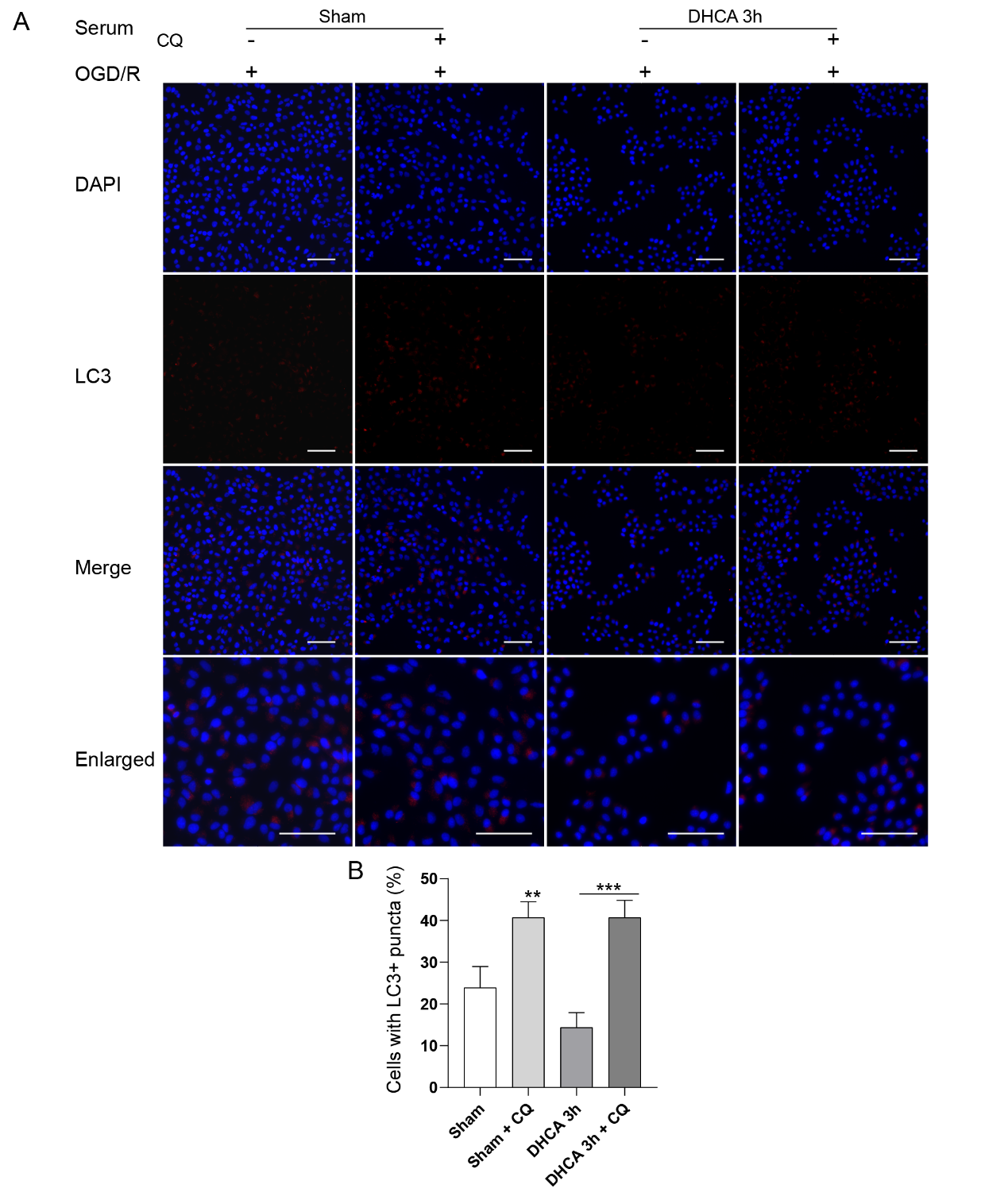


**Online Figure V. (A)** Compared with 16HBEs + Sham (16HBE cells treated with the serum of group Sham rats for 24 hours), immunofluorescence staining of 16HBEs + (Sham + chloroquine (CQ)) showed that the expression level of LC3-II was significantly increased; similarly, the level of 16HBEs + (DHCA 3h + CQ) was higher than that of 16HBEs + DHCA 3h. Rapamycin increased the level of LC3-II. The enlarged picture is shown at the bottom (Bottom panel). (Scale bar=100 μm). Red, LC3-positive cells; blue, DAPI-stained nuclei. **(B)** Quantitative analysis.


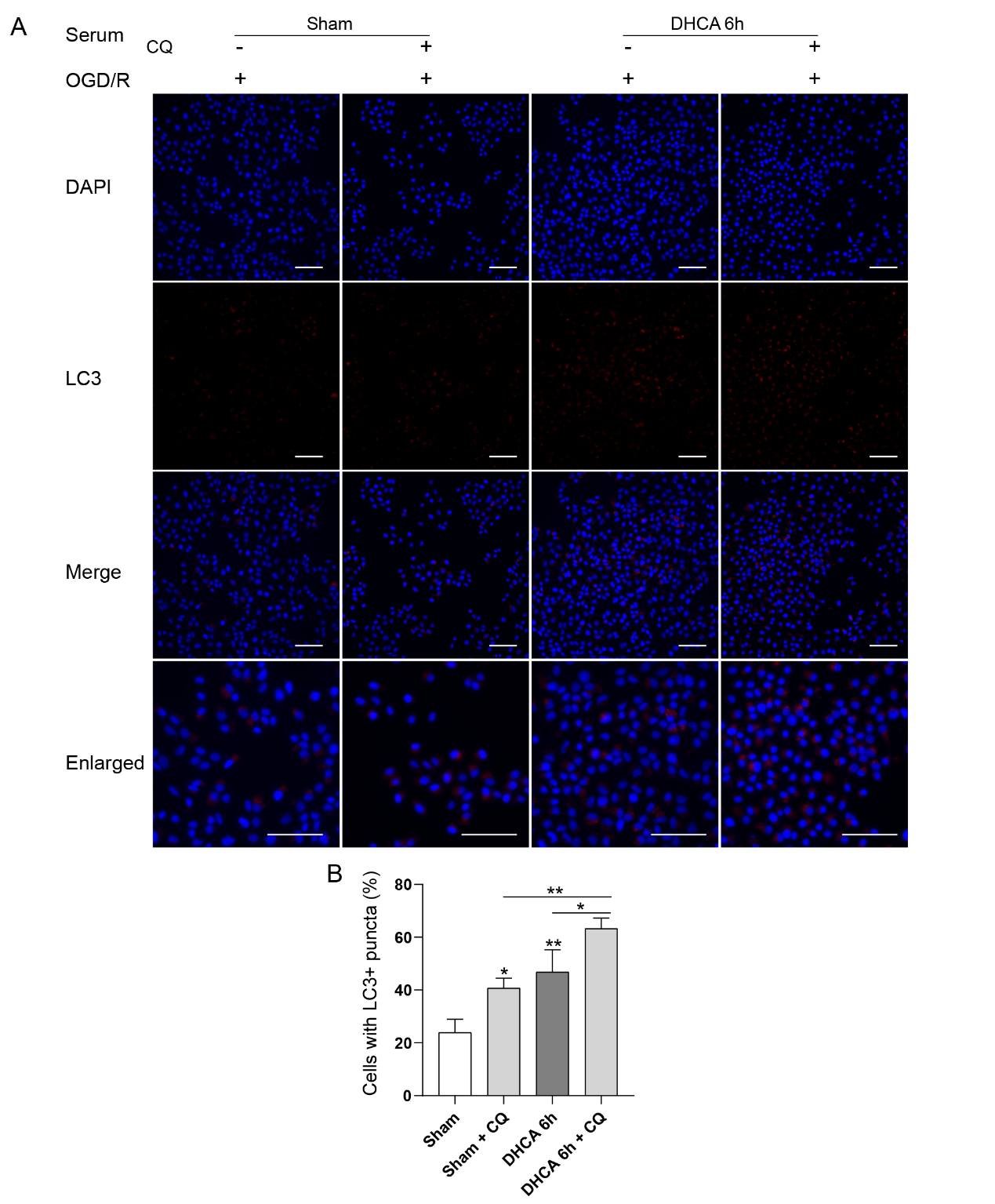


**Online Figure VI. (A)** Compared with 16HBEs + Sham (16HBE cells treated with the serum of group Sham rats for 24 hours), immunofluorescence staining of 16HBEs + (Sham + CQ) showed that the expression level of LC3-II was significantly increased; similarly, the level of 16HBEs + (DHCA 6h + CQ) was higher than that of 16HBEs + DHCA 6h. Rapamycin increased the level of LC3-II. The enlarged picture is shown at the bottom (Bottom panel). (Scale bar=100 μm). Red, LC3-positive cells; blue, DAPI-stained nuclei. **(B)** Quantitative analysis.


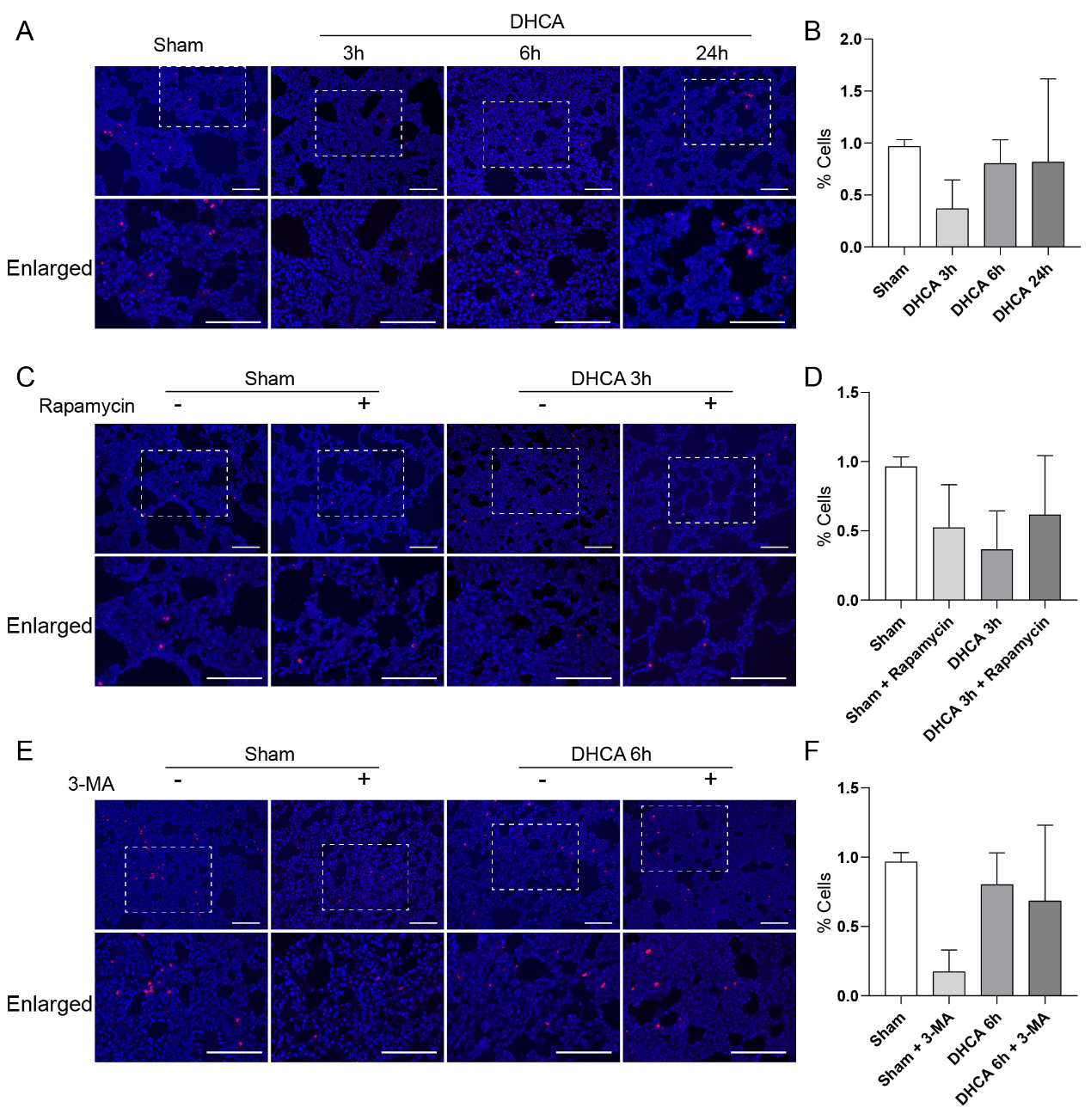


**Online Figure VII.** TUNEL assay of lung tissues. **(A, C, and E)** Lung sections were obtained from group rats. The average number of TUNEL stained cells in each group accounted for less than 1%, and there was no statistical difference between the groups. The enlarged picture is shown at the bottom (Bottom panel). (Scale bar=100 μm). Red, TUNEL positive cells; blue, DAPI-stained nuclei. **(B, D, and F)** Quantitative TUNEL analysis.
